# Interpersonal relationships as coping mechanisms during bed rest: a thematic synthesis literature review

**DOI:** 10.3389/fpsyg.2024.1501113

**Published:** 2025-01-07

**Authors:** Ana Cikač, Saša Pišot

**Affiliations:** ^1^Institute for Kinesiology Research, Science and Research Centre Koper, Koper, Slovenia; ^2^Faculty of Social Sciences, University of Ljubljana, Ljubljana, Slovenia

**Keywords:** bed rest, social connectedness, interpersonal relationships, coping mechanisms, thematic synthesis, literature review

## Abstract

Bed rest (BR) studies are primarily designed to investigate the effects of weightlessness on the human body, but they are also used to study the effects of physical inactivity. For this purpose, participants are typically recruited from the general population without requiring specialized training, which contrasts with the selection process for cosmonauts. The BR study environment is often characterized as highly stressful, highlighting the importance of understanding coping mechanisms and adaptation strategies among participants, as well as the role of their daily interactions. This review aims to determine whether interpersonal relationships and the concept of social connectedness (SC) have been explored within the context of BR studies. For the search strategy, the definition of exclusion criteria, and the initial screening, PRISMA 2020 statement was followed. The PEO framework was used to generate keywords, and thematic synthesis was applied for data extraction, analysis, and synthesis. An initial search did not uncover any studies examining the concept of SC in the context of BR as an environment with extreme conditions, suggesting that SC in this context has not yet been investigated. While findings of thematic synthesis indicate that interpersonal relationships play a significant role in coping with and adapting to the extreme conditions of BR studies. As results showed these relationships can have both positive and negative effects. Interpersonal relationships also serve as a crucial support mechanism among participants. Additionally, how participants make sense of their involvement in such studies remains underexplored, and further research in this area is recommended in the discussion.

## Introduction

In the 1990s, studies on simulating weightlessness, known as bed rest (BR) studies, began to investigate the effects of such an environment on the human body in space (Jost, 2008). Over time, various forms of BR studies have been developed, differing in terms of duration, type of microgravity simulation (such as head-down tilt, hypoxic, horizontal), and population parameters. However, BR studies are now considered the gold standard for investigating the effects of weightlessness on the human body, particularly the so-called head tilt, in which the head is tilted by 6° while lying down (Harris et al., 2022). Otherwise, BR studies also become commonly used to simulate extreme physical inactivity (PI) and investigate its effects on the human body (Pišot et al., 2008a) such as the decline in muscle mass and strength (Pišot et al., 2008b; Marušič et al., 2021), or the fluctuations in blood glucose concentrations (Trim et al., 2023). The importance of conducting BR studies to investigate PI has increased significantly (Pišot et al., 2008a), particularly as insufficient physical activity has become a global health problem. At the same time, sedentary behavior has risen globally (WHO, 2020) contributing to long-term negative effects on the human body (Park et al., 2020) and even increased risk of mortality from various causes (Van Der Ploeg, 2012). However, there is an important distinction between BR studies focusing on cosmonauts and the simulation of the space environments, and those aimed at addressing PI to apply findings to the general population.

Since the 1990s, parallel studies have been conducted alongside investigations into the effects of weightlessness on physiological factors. These studies primarily focus on psychological factors related to extreme environments and group dynamics. For example, they include simulations of extreme environments in Antarctica, studies in real space environments on international space stations (ISS), and, to a lesser extent, BR studies. However, they are primarily used to investigate the psychological factors and group dynamics of cosmonauts in order to put together the best possible team for a real space mission (Sandal, 1998; Palinkas, 2003; Pagnini et al., 2023). These studies are being conducted by the National Aeronautics and Space Administration (NASA), European Space Agency, and Institute for Biomedical Problems (Seaton et al., 2009; Pagnini et al., 2023; Supolkina et al., 2023; NASA Flight Analogs, 2024).

Extremely isolated environments have been found to be particularly challenging for individuals to adapt to, both in terms of the new environment and interaction with other individuals in that setting. Therefore, such environments are defined as highly stressful, and various deviant behaviors such as aggression, may develop within groups toward other participants. Personality traits also play an important role, for example, individuals with high motivation tend to adapt more easily to an extreme environment (Sandal, 1998). As early as 2003, Palinkas noted in a review article that: “Members of expeditions with low social coherence report significantly more depression, anxiety, and anger than those in expeditions with high social coherence.” (Palinkas, 2003, p. 353).

Further studies up to the present day have been conducted on psychological factors and group dynamics, focusing mainly on cosmonauts and various forms of space expeditions (Pagnini et al., 2023). For example, a recent study by Supolkina et al. (2023) analyzed conversations between cosmonauts on the ISS and confirmed that the form of communication between crew members depends on the level of daily workload. Positive communication also means appropriate support for co-participants and serves as a coping mechanism in an extreme and isolated environment.

Participants in BR studies, that focus on simulating PI, are predominantly recruited from the general population, as the purpose is not necessarily to apply the results to cosmonauts, but rather, particularly in the case of studying PI, to the general population. This is an important distinction, as participants from the general population have no special training like cosmonauts, nor are they given specific instructions on how to communicate or interact with other participants or personnel. For example, standards have already been established for space missions on the ISS: “standard human behavior and performance competency framework for crewmembers” (Pagnini et al., 2023, p. 2), which provides guidelines for appropriate behavior and delegates responsibilities to maintain cohesive relationships between crewmembers. This underscores a significant gap in research on the role of interpersonal relationships (IR) in adapting to the challenging conditions of BR studies, particularly those that simulate extreme PI and recruit participants from the general population.

The concept of social connectedness (SC), as defined by Lee and Robbins, represents a specific type of belongingness or the lack thereof, experienced as a sense of SC. It is grounded in the psychoanalytic theory of self-psychology. The concept of SC emphasizes the independent individual in relation to others and is distinct from the classical definitions of belongingness or loneliness. It reflects the feeling or recognition of interpersonal connectedness with the social world as a whole, placing the independent individual at the forefront (Lee and Robbins, 1995, 1998). To measure SC, a standardized and validated questionnaire (SCS-Original) was developed. Later, a reverse-scored version (SCS-R) was created to assess psychological distress and dysfunctional interpersonal behaviors (Lee et al., 2001). Given the primary definition of the concept of SC, it would be valuable to explore it in the context of BR studies, as the unique environment of these studies can be perceived as a form of isolation, potentially altering the sense of SC.

In later studies, the concept of SC was also applied to the study of group dynamics, focusing on the sense of interpersonal connectedness with others within the same group. In a study by Lee et al. (2008), SC was found to mediate how individuals make sense of their social experiences, self-organize, and engage in behaviors that strengthen IR and enhance subjective well-being. Furthermore, a scale to measure SC in groups was developed (Lee and Davis, 2000). Based on these findings, it would be valuable to explore the concept of SC in relation to IR, particularly its role in coping with and adapting to the extreme environment of BR studies. These BR studies are typically structured so that participants are divided into groups or assigned to specific rooms, where they remain during the entire lying phase of the experiment.

Therefore, this literature review focuses on the research question: What role do interpersonal relationships (IR) play in coping with and adapting to the extreme conditions of BR studies among BR participants recruited from the general population?

This thematic synthesis literature review aims to gather scientific evidence to address the research question: What role does SC play in participants’ adaptation to the extreme conditions of BR studies? Specifically, this review seeks to determine whether IR and the concept of SC have been explored within the context of BR studies. The purpose of this literature review is also to identify areas for further research, focusing on the topics mentioned above.

## Materials and methods

After a brief scan of the literature on conducting literature reviews with a focus on qualitative methods in the social sciences (Petticrew and Roberts, 2006; Thomas and Harden, 2008; Bettany-Saltikov, 2010a, 2010b; Xiao and Watson, 2019; Page et al., 2021), the PRISMA 2020^1^ statement (Page et al., 2021) was followed to guide search strategy, define exclusion criteria and conduct an initial screening. The PEO^2^ framework (Bettany-Saltikov, 2010a) was used to generate keywords. For data extraction, analysis, and synthesis, thematic synthesis was applied (Thomas and Harden, 2008), which includes content analysis.

The literature search was conducted using three databases: Web of Science, Google Scholar, and Scopus. The first two databases have a global orientation, while the latter focuses on the European research area. These databases were selected based on the criterion of “broad coverage”, aiming to encompass both natural and social science perspectives on the topic.

Used keywords are listed in Table 1. For the selection and organization of keywords, the PEO framework was used, which is particularly suitable for formulating review questions and associated keywords for qualitative research (Bettany-Saltikov, 2010a).

**Table 1:** Combination of keywords based on PEO model.

Population	AND exposure	AND Individual response* - outcome	AND Term
1. Males	4. bed rest	6. coping	10. social connectedness
2. Young adults	5. simulating microgravity	7. resilience	11. interpersonal relationships
3. Older people		8. adaptation	12. coping
		9. mood	13. mood
			14. adaptation
			15. resilience
Combine 1–3 using OR	Combine 4–5 using OR	Combine 6–9 using OR	Combine 10–15 using the OR

OR = search for scientific articles that contain one of the selected words or phrases: AND = search for scientific articles that contain all of the selected words or phrases. Adapted from Bettany-Saltikov (2010b).

The keywords were entered into the search engines as represented in Table 1. The words were entered vertically in groups of 1–3, 4–5, and so on, using the conjunction OR, and horizontally, the word groups were entered using the conjunction AND. After entering the keywords, the following filters were applied: time frame (1990–2024), demographics [male, human, young adult, older adults (65+), and study type (clinical trial)]. The time filter (1990–2024) was selected based on a preliminary review of the literature, which identified the 1990s as the starting point for significant BR research (Jost, 2008).

After the initial search, 178 scientific articles were identified. From this, 24 duplicates were found, so 12 articles were eliminated. Therefore, a total of 166 articles were included in the initial screening.

For the initial screening, the online and mobile application for systematic reviews Rayyan (Ouzzani et al., 2016) was used, while applying the following exclusion criteria:

1. Not a BR study but articles focusing on other forms of microgravity simulation (*n* = 54).
2. Type of BR study: articles on specific types of BR, such as hypoxic BR, sedentary BR, pregnancy BR, or dry immersion (*n* = 12).
3. Field of interest. Articles in fields unrelated to our focus, including medicine, microbiology, neuroscience, and clinical psychology *(n* = 81).
  - Population Exclusion (*n* = 11): Articles involving only female participants, as the Google Scholar database does not provide a population filter (*n* = 4), articles that involve non-human subjects and articles that examined the effects of weightlessness simulation on animals, such as rats or mice (*n* = 7).

The initial inclusion criteria encompassed the following conditions: (1) The form of BR (horizontal or head-down tilt (6°); (2) The field of interest (psychology or sociology or study of IR); (3) The population studied (human), and (4) Sex (male).^3^

**Figure 1:**
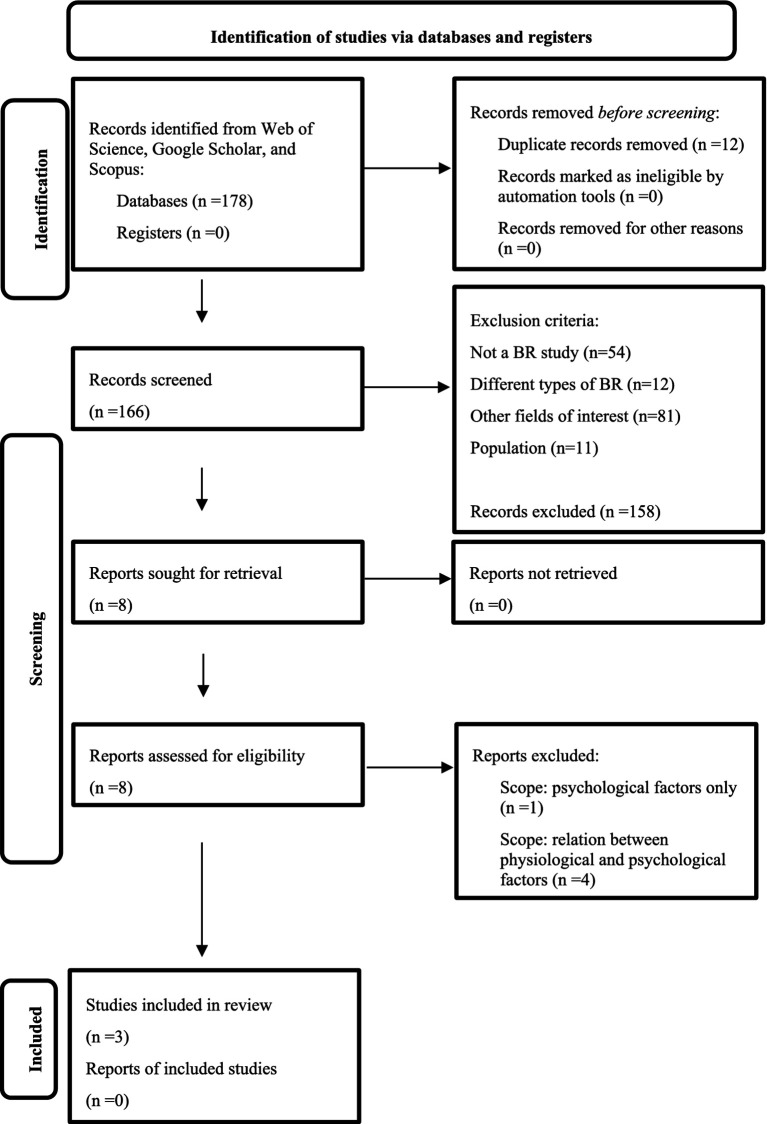
The process of searching and selecting studies for the present thematic synthesis literature review. Adapted from PRISMA 2020 (Page et al., 2021).

A total of eight studies were evaluated for the eligibility assessment, and the final inclusion criteria for the study selected for the review were established, resulting in three studies being included (*n* = 3). These studies, which are summarized in Table 2, met the following inclusion criteria: (1) They measure or observe SC, IR, or social support, and (2) Their primary focus was on social interaction or social relationships. A figurative representation of the entire process of searching and selecting studies is illustrated in the PRISMA 2020 flow diagram for systematic reviews (Page et al., 2021), found in Figure 1.

**Table 2:** Basic characteristics of the studies selected for the final review.

	Authors	Aim/objective	Form of BR	Duration of BR (days)	Age (years)	Sex	Number of participants	Method
1	Weiss and Moser (1998)	To explore interpersonal relationships in insolation and confinement, simulated as BR study	Head-down tilt 6°	42	25–35	M	8	Non-participant observation
2	Wu et al. (2023)	To explore change in personal values in a 90-day BR study	Head-down tilt 6°	90	30–42	M	36	Questionnaire non-participant observation
3	Ishizaki et al. (2004)	To explore the interaction produced by the game in decreasing participants’ psychological stress and mental health in a BR environment	Head-down tilt 6°	20	20–26	M	12	Questionnaire non-participant observation

Two of the studies employed a quantitative questionnaire method (Ishizaki et al., 2004; Wu et al., 2023). Additionally, Wu et al. (2023) used the observation method, while Weiss and Moser (1998) relied solely on observational methods, we therefore had to include data obtained using both quantitative and qualitative methods in the analysis. Furthermore, all studies included in the final review interpreted their results using a combination of methods and presented their findings in both, the results and discussion sections, necessitating the extraction of data from the discussion part of the text. Consequently, the content analysis method was chosen for this review. Data analysis was conducted using MAXQDA 2022 (2021). The evaluation or validation of the quantitative methods used in the articles was therefore not part of the analysis.

For data extraction and synthesis, we followed the protocol for thematic synthesis as outlined by (Thomas and Harden, 2008). This approach was well suited to the nature of our research question and the qualitative methodology used in the studies included in the final review. According to Thomas and Harden (2008), thematic synthesis involves three stages:(1) coding the text, (2) developing descriptive themes, and (3) generating analytical themes.

In the first stage, we coded the text of the included studies, focusing on the results and discussion sections, meaning only these two parts were coded systematically. Any practice related to IR among participants was coded. A total of 9 codes, with 61 coded segments emerged from this initial coding process. The original 9 codes were then expanded by adding either a positive or non-positive connotation to each. Two sets of identical codes were obtained, allowing both positive and negative connotations to be encoded for each code in the text. This was done to ensure that the potential positive or negative role of IR in adapting to the extreme conditions of the BR study was not overlooked.

In the second stage, the codes from the coding system were analyzed. First regrouping them into the original 9 coding categories. Codes were then reviewed to identify similarities and differences. During this process, the initial 9 codes were merged to identify common descriptive themes, resulting in the creation of 5 main descriptive themes: (1) coping and adaption to new situations (extreme environment), (2) support from others, (3) personal values, (4) resilience, and (5) making sense of participation.

**Figure 2:**
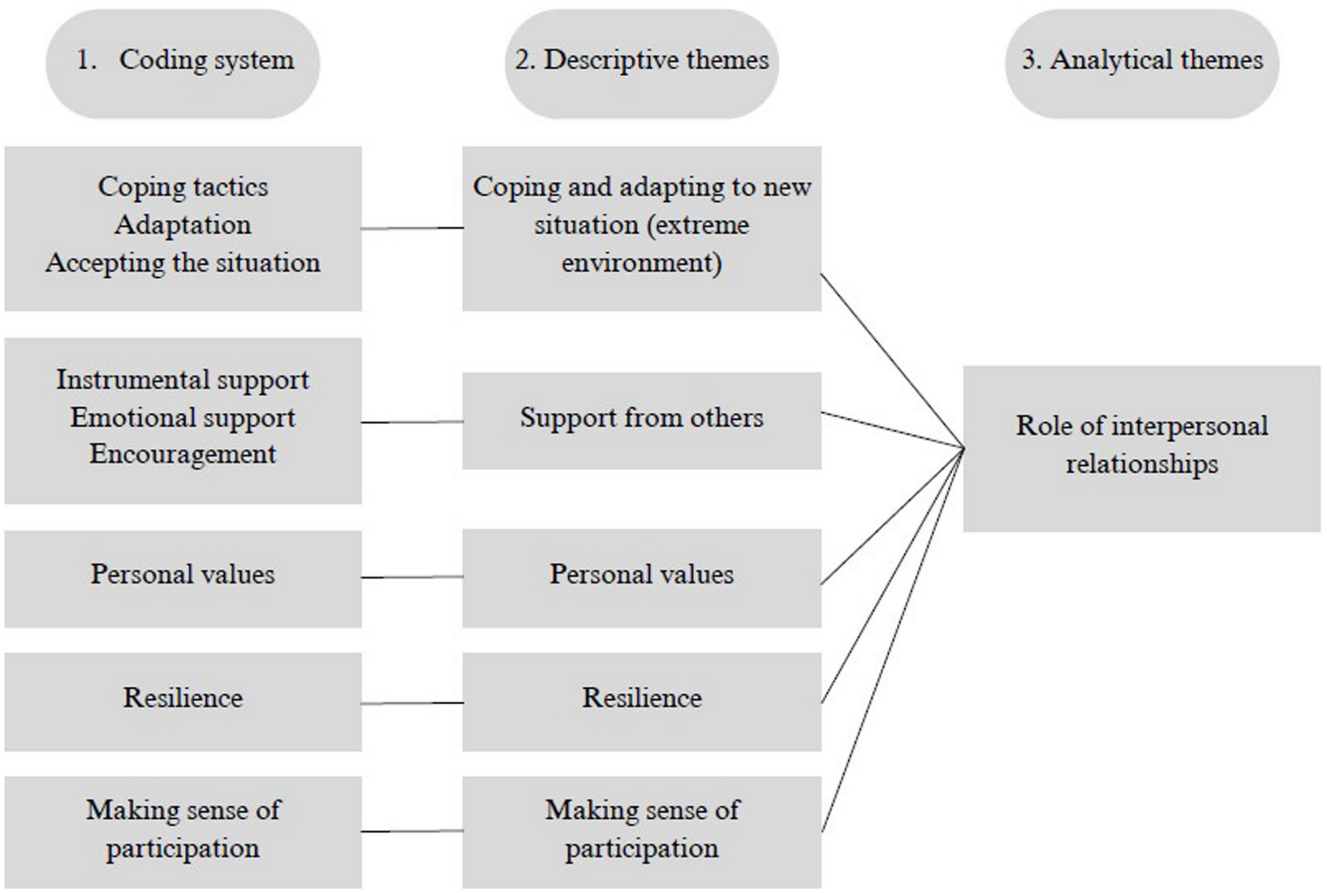
Process of data coding and analysis. For creating using Canva (2024).

In the following third stage, the main focus was on generating analytical themes. This was the most challenging part of the process, during which descriptive themes were compared with the research question. Additionally, concepts related to the tools participants might use to cope with extreme situations were examined. At this point, it was also crucial to consider the significance of IR within the context of BR as an extreme environment, which led to the development of one analytical theme: *the role of interpersonal relationships*. The process of data coding and analysis is illustrated in Figure 2.

## Results

### Results of the analysis of coding coverage within an individual text

First, a coding coverage analysis was conducted to identify differences in coding distribution within each text, providing information on which codes appear more often and which appear less often within each text. The coded segments of the coding system are distributed differently in each text, depending on the research focus of the respective study, which is not a surprising result. A closer analysis of the coding coverage within each text reveals that the first study by Weiss and Moser (1998), has the highest coding coverage for code *coping tactics* (66% of the coded text) and the lowest for code *personal values* (19% of the coded text). The second study from Wu et al. (2023) showed that coded coverage has also been highest for code *personal values* (32% of the coded text) and code *coping tactics* (32% of the coded text) with the lowest coverage for code *encouragement* (6% of the coded text). The third study (Ishizaki et al., 2004) has the highest coded coverage of coded text for code *adaptation* (67%) and the lowest for *encouragement* (19%).

### Results of the analysis of coding coverage between texts

Next, a coding coverage analysis was conducted to compare coding coverage across texts, focusing on each specific code. This provided information on the coverage of individual codes of the coding system across all texts combined. When analyzing the coding coverage between the texts of the reviewed studies, the results show that the code *coping tactics* occur most frequently in the first study (66% of the coded text), followed by the third study (46% of the coded text) and then the second study (32% of the coded text). For the code *adaptation*, the highest coding frequency is in the third study (67%) while the lowest is in the second study (21%). The coded and derived theme *making sense of participation* is present only in the second study.

### Results of the analysis of coding distribution across all text combined

After merging codes into descriptive themes, an analysis of coding distribution was conducted. In the first stage, the results of coding distribution from the MAXQDA program were extracted. In the second stage, codes were merged into descriptive themes, and an analysis of coding distribution based on code frequencies was performed.

**Figure 3:**
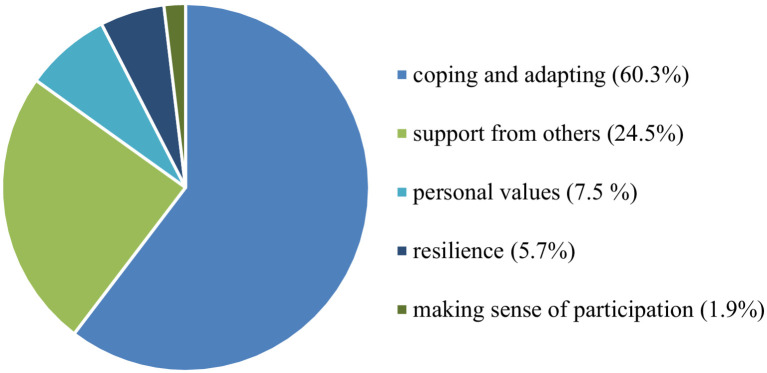
Frequency of descriptive theme distribution in all three studies, presented in percentages.

The overall analysis reveals that the most common descriptive theme to emerge is *coping and adapting* to a new situation (extreme environment) (60.4%), followed by *support from others* (24.5%), *personal values* (7.5%), *resilience* (5.7%), and finally, the *making sense of participation* (1.9%). The results are shown in Figure 3. Frequencies are represented as proportions in this figure, with each frequency expressed as a percentage of the total. This approach facilitates a clearer comparison of the relative occurrence of each descriptive theme, providing insight into their distribution and importance within the overall dataset.

**Figure 4:**
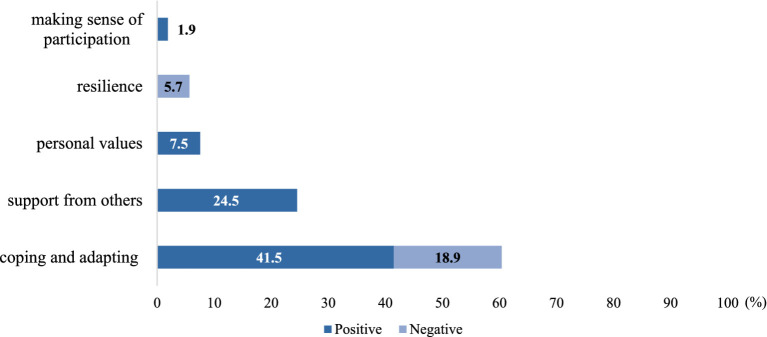
Distribution of positive and negative connotations of descriptive themes.

The results also showed that the role of IR can be either positive or negative concerning some descriptive themes, meaning that certain forms or practices encourage, while others discourage IR. This distribution of descriptive themes is shown in Figure 4. In general, IR are defined as positive in relation to all five descriptive themes, with a coding frequency of 75.5%, whereas negative relationships have a coding frequency of 24.5%.

Some descriptive themes have only negative connotations, such as *resilience* (5.7%), while others have only positive ones, such as *personal values* (7.5%) and *support from others* (24.5%). Notably, the descriptive theme of coping and adapting has both positive (41.5%) and negative (18.9%) connotations, with the positive connotation being the most prevalent.

## Discussion

The concept of SC in the context of BR as an extreme condition and environment, which was the primary interest, was not addressed by any of the reviewed studies. Furthermore, the initial search that was conducted did not reveal any studies on this topic, suggesting that the concept of SC in the context of BR has not yet been explored. Therefore, the research focus was broadened to examine IR within the BR environment. This broader focus could provide a similar overview of the research field, centered on the relationships between participants and the role these relationships play in participation and adaptation to the BR environment.

The results are organized into three main themes that integrate both descriptive and analytical aspects for discussion purposes: *coping and adapting to a new situation (extreme environment)*, *support from others*, and *making sense of participation*. Following the analysis, we decided to include in the discussion the descriptive themes most relevant to addressing the research question. Specifically, we highlighted the two descriptive themes most prominently represented in the coding distribution (see Figure 3). Additionally, we considered it important to include a descriptive theme that was the least represented in the coding distribution. This choice underscores its limited exploration in the analyzed articles and highlights opportunities for further research in this area.

### Coping and adapting

The most common descriptive theme that emerged in the analysis is *coping and adapting to a new situation (extreme environment)*, which shows us that this was the most frequently investigated theme in the studies examined, even if this was not necessarily the main objective of the studies.

All studies define BR as an extreme environment that increases psychological stress for participants (Weiss and Moser, 1998; Ishizaki et al., 2004; Wu et al., 2023). This is consistent with findings from studies on social relationships and psychological factors in simulating microgravity during polar expeditions and space simulations (Sandal, 1998; Palinkas, 2003; Pagnini et al., 2023). However, there is still too little research on the role of IR in coping with and adapting to extreme situations in specific environments of BR studies.

Based on the present review, it can be concluded that IR plays a dual role in *coping and adaptation* with both positive and negative effects. This suggests that participants sometimes seek companionship, while at other times they prefer isolation, indicating the complexity and multi-directional nature of social relationships. These relationships are crucial in the adaptation and coping process of individuals in extreme situations and in the new environment of BR, as they can either increase or decrease the psychological burden of the new situation for the participants’ (Weiss and Moser, 1998; Ishizaki et al., 2004; Wu et al., 2023).

Different forms of IR can either enhance the positive aspects or amplify the negative aspects of interactions, offering valuable insights for the design and organization of experiments. For example, collaborative activities (such as playing a game together) can foster positive IR (Ishizaki et al., 2004). This aligns with findings from Palinkas (2003)'s review article on human behavior in isolated and confined environments, particularly in Antarctica. However, further research is needed to define and identify additional forms of interpersonal activity and to compare these with variables such as gender, age, and the cultural and social backgrounds of participants.

During the study, participants sought isolation at least as much as they sought interpersonal contact (Weiss and Moser, 1998). This finding underscores the importance of closely observing IR during the implementation of a BR study. The 1998, Weiss and Moser study also found that one of the individuals stood out from the other subjects due to an extreme tendency toward withdrawn behavior and a preference for solitude throughout the experiment, engaging in solitary activities like reading, inactivity, etc. On the 29th day of BR, the subject’s roommate withdrew from the experiment, making him the only participant who did not complete it (Weiss and Moser, 1998). This underscores the importance of IR in coping with new and extreme situations and highlights the need for a deep understanding of the complex dynamics of these relationships. Such an approach could contribute to better study designs, including the considerations for space arrangements and participant selection, ultimately helping to alleviate the stress experienced by participants.

This approach aligns with NASA’s Human Research Program, which uses the Flight Analogs Project to study the physiological and psychological effects of isolation and confinement on humans (NASA Flight Analogs, 2024). The study *Behavioral and Psychological Issues in Long-Duration Head-Down Bed Rest* by Seaton et al. (2009) suggested psychological assessment of potential participants during the initial screening phase of a study and the training of staff to provide appropriate psychological support to study participants. However, it is important to note that these studies are focused on applying the results to space missions and are mainly concerned with the physiological and psychological effects on the space crew or cosmonauts (Seaton et al., 2009; NASA Flight Analogs, 2024).

Additionally, BR studies are conducted to investigate the consequences and changes of weightlessness as a simulation of PI (Pišot et al., 2008b; Marušič et al., 2021). When recruiting subjects for these studies, it may not be necessary to focus on specific psychological characteristics important for cosmonauts. Instead, the focus could shift to exploring IR and the social environment. This approach could help identify additional forms of interpersonal activity that enhance the research’s effectiveness and alleviate stressors for participants.

### Support from others

*Support from others* is a descriptive theme that appears only in positive connotation to IR, through reviewed studies. This suggests that IR among participants can offer support and help alleviate the stressful circumstances of participation. However, this topic warrants closer examination, as studies simulating isolated and extreme conditions have shown that such stressful environments can also trigger deviant behaviour toward co-participants, as highlighted in Palinkas’s literature review (Palinkas, 2003).

The BR study by Weiss and Moser (1998) found that support from others can be both emotional and instrumental. In instrumental support, participants seek information from co-participants, compare themselves to others in the same situation, and use this information as guidance on how to navigate in this new, particular situation. This process is defined as *social comparison* in the study. On the other hand, the search for empathy, described as an *emotional comparison* was equally important as participants sought empathy: “to feel reassured and reinforced in the understanding that the stressful situation was shared by everyone.” (Weiss and Moser, 1998, p. 246).

Of the studies analyzed in this review, only the aforementioned study by Weiss and Moser defines these two forms of support. However, elements of *support from others* are identified in the studies by Wu et al. (2023) and Ishizaki et al. (2004), though this was not the primary focus of these studies. In Ishizaki et al.’s (2004) BR experiment implementing a game created an important connection between participants by giving them a starting point for conversations, which then led to broader discussion and mutual support. Although Wu and colleagues' (2023) study primarily focuses on individual aspects of participants’ involvement, it mentions that the presence of a co-participant in the room played a crucial role in fostering support between subjects. The study also reported that participants described themselves as a strong group after the experiment. Additionally, it was found that even though the importance of individual personal values increased during the experiment, the personal value of “social interest” was consistently rated as the most important by participants from the beginning to the end of the study, remaining stable throughout (Wu et al., 2023). In the two studies mentioned above, there is insufficient information or, data to clearly differentiate between instrumental and emotional support. However, we can conclude that *support from others* plays a crucial role in participants’ IR across all the reviewed studies.

### Making sense of participation

The last descriptive theme in the discussion is the one that appears the least frequently in the analysis, as already indicated in the results (see Figure 3). This suggests that participants’ conceptualization of participation was the least researched among the analyzed studies. It is important to note that this topic was not the primary focus of any of the studies; rather it was only indirectly addressed in the study on personal values by Wu et al. (2023). In their study, participants’ conceptualization of participation, particularly their persistence in completing the study, was explained as a means of fulfilling societal and environmental expectations. This perspective may be linked to a particular aspect of Chinese culture, which emphasizes social responsibility and encourages cooperation within the community (Wu et al., 2023).

The finding that conceptualizing participation appears the least researched descriptive theme is important, especially for understanding participation in the study from the participants’ perspective. To capture firsthand experiences of participation, methods such as semi-structured interviews and participant observation could be employed. This approach could help address several questions, such as what participation in the study means to the participants, how they navigate the extreme circumstances during the study, and what motivated them to participate. Gaining insights into these aspects could lead to a better understanding of the participants’ perspectives, ultimately contributing to improved organization and execution of BR studies.

It is important to recognize that BR studies also recruit individuals without specialized training, such as cosmonauts. These participants typically enter the study with different intentions and motivations compared to trained cosmonauts, which can result in vastly different experiences from their perspective.

To better understand this perspective, Interpretative Phenomenological Analysis could be used as a method. The essence of interpretative phenomenological analysis lies in: “understanding personal lived experience and thus with exploring persons’ relatedness to, or involvement in, a particular event or process (phenomenon).” (Smith et al., 2022, p. 35). As Smith et al. (2022) explain, interpretative phenomenological analysis involves the exploration, description, and interpretation of the everyday reality of the lived experience as our participants perceive and make sense of it.

Using this approach, an important contribution could be made to a more detailed investigation of the descriptive theme of *making sense of participation*, which was found to be insufficiently researched in the analysis.

## Limitations

Given that the literature review encompasses the fields of sociology, psychology, and medicine, the PRISMA 2020 statement was employed for the initial search which is a standardized form for systematic reviews. However, the PICO framework was not followed for formulating the research question; instead, the PEO framework, most commonly used in qualitative studies (Bettany-Saltikov, 2010a), was applied. Additionally, the PRISMA 2020 guidelines for synthesis were not strictly adhered to, as they were considered better suited for qualitative synthesis. This adaptation may have resulted in some inconsistencies within the literature review.

As the goal was to look beyond the primary content of the studies; a quantitative assessment and synthesis of study results following the systematic review approach was not chosen. Instead, the thematic synthesis literature review was intended to evaluate the results and conclusions of research intersecting the topics of BR studies and IR. To this end, a thematic synthesis (Thomas and Harden, 2008) was applied, allowing the interpretations of the results - a key aspect of this thematic synthesis literature review. The studies included in thematic analysis encompassed not only questionnaire data but also observational findings, whose interpretation is essential for identifying descriptive themes. However, this approach may have limitations in terms of the reliability of the study results, as the evaluation of quantitative methods was not the primary focus.

A significant challenge in synthesizing the results is the variability in the types of BR studies. Although studies involving BR with a 6° head-down tilt were included in the final review and synthesis, they differed in the number of participants and room assignments. For instance, in the study by Weiss and Moser (1998), 8 men were recruited, who slept in twin rooms, with participants choosing their roommates themselves. In contrast, the study by Ishiaki and colleagues (2004) recruited 12 men, 6 of whom shared a room, and who already knew each other since the invitation to participate was sent exclusively to the student population. Given the different socio-cultural contexts, population differences (including the age of the participants), and different forms of BR studies that create different and unique extreme circumstances and experiences, comparing results across studies is challenging, representing a significant drawback.

As most of the studies identified in the original search focused primarily on male participants, this literature review also focuses on the male population, with an emphasis on recruitment from the general population. However, this may impose certain limitations as the literature review should also include female participants to account for possible sex differences. Female participants are often recruited less frequently for BR studies than male participants. One explanation for this could be that BR studies are primarily designed to investigate the effects of weightlessness on the human body on Earth (Jost, 2008), combined with the statistical fact that the vast majority of space travel is conducted by male astronauts and cosmonauts (Roberts, 2022). More frequent inclusion of female participants in BR studies could provide valuable insights into sex and gender differences in perception, IR, and coping mechanisms in the BR environment.

## Conclusion

Based on the research question and criteria, the initial search revealed a limited number of studies that thematically explore the intersections between BR as an environment of extreme conditions and the role of IR in coping and adapting to these environments. Furthermore, the studies analyzed differ in terms of format, methods, participant groups, and sociocultural background, making comparison challenging. Further research is needed to investigate these differences in more detail.

Nonetheless, this thematic synthesis literature review has shown that the concept of SC has not yet been explored in the context of BR as an environment with extreme conditions. This suggests the need for further research. Further research is also needed to examine the first-hand experiences of BR participants to gain insights into their perceptions of an extreme environment and the role of IR as a coping mechanism.

This approach could contribute to a better understanding of the relationship dynamics during the study and ultimately support the organization and execution of future BR studies. By gaining precise knowledge of the factors or interventions that reduce stress among participants, we can conduct BR studies simulating extreme PI more effectively. This, in turn, creates a more optimal environment for studying the physiological effects of extreme PI on the human body. Additionally, understanding how to design supportive study environments can ease participation for the individuals involved. Finally, insights into the importance of interpersonal relationships in controlled hospital-like settings hold significant value for the field of medicine. A deeper understanding of interpersonal dynamics could help address potential complications arising during the hospitalization of individuals placed together in shared hospital rooms.

## Data Availability

Publicly available datasets were analyzed in this study. This data can be found here: Articles that were included in the review and analysis after the initial search, were obtained from publicly available databases. The original set is kept in the Ryyan program of the first author and accessible upon request.

## Footnotes

^1^The PRISMA 2020 statement stays for The Preferred Reporting Items for Systematic reviews and Meta-Analyses (PRISMA), and contains guidelines for authors to help them conduct and report systematic literature reviews (Page et al., 2021). Since being established in 2009, it has been massively used by scientists in natural sciences and has become some of a golden standard for conducting systematic literature reviews and meta-analyses.

^2^PEO is the abbreviation for population, exposure and outcome. It is used to determine the components of the research question and is normally used for qualitative review studies (Bettany-Saltikov, 2010a).

^3^This literature review serves as a foundation for further research using the example of a conducted BR study that included male participants. As a result, this review applies specific exclusion criteria for female participants. However, in the absence of these particular circumstances, it would be appropriate to include female participants as well, as discussed in detail in the limitations section.
